# Dynamic predictability and activity-location contexts in human mobility

**DOI:** 10.1098/rsos.240115

**Published:** 2024-09-04

**Authors:** Bibandhan Poudyal, Diogo Pacheco, Marcos Oliveira, Zexun Chen, Hugo S. Barbosa, Ronaldo Menezes, Gourab Ghoshal

**Affiliations:** ^1^ Department of Physics & Astronomy, University of Rochester, Rochester, NY, USA; ^2^ Department of Computer Science, University of Exeter, Exeter, UK; ^3^ GESIS—Leibniz Institute for the Social Sciences, Cologne, Germany; ^4^ The University of Edinburgh Business School, Edinburgh, UK; ^5^ Department of Computer Science, Federal University of Ceará, Fortaleza, Brazil

**Keywords:** complex systems, information theory, human mobility

## Abstract

Human travelling behaviours are markedly regular, to a large extent predictable, and mostly driven by biological necessities and social constructs. Not surprisingly, such predictability is influenced by an array of factors ranging in scale from individual preferences and choices, through social groups and households, all the way to the global scale, such as mobility restrictions in response to external shocks such as pandemics. In this work, we explore how temporal, activity and location variations in individual-level mobility—referred to as *predictability states*—carry a large degree of information regarding the nature of mobility regularities at the population level. Our findings indicate the existence of contextual and activity signatures in predictability states, suggesting the potential for a more nuanced approach to estimating both short-term and higher-order mobility predictions. The existence of location contexts, in particular, serves as a parsimonious estimator for predictability patterns even in the case of low resolution and missing data.

## 1. Introduction

The understanding of the mechanisms governing human travel behaviour is crucial to a variety of domains such as epidemic modelling [1–5], transportation [6–9], national security [10], urban planning [11–18] and a host of other applications [19]. Human mobility trajectories have been shown to exhibit statistical regularities at multiple scales [20,21], despite the inherent complexity that exists in the available choices for the routes of their daily travels [22,23]. These regularities are rooted in an array of social, spatial and temporal mechanisms, leading to visitation patterns being highly regular [24]. Indeed, it has been shown that a perfect algorithm can predict, with between 70% and 90% certainty, an individual’s future location given their prior location visits [25], depending upon the spatio-temporal granularity of observations [26,27]. In practice, however, the predictive task has proven to be more challenging than what the high predictability could suggest. Current predictive models have been achieving performances ranging from 10% to 15% on similar mobility datasets [28–30].

Moreover, human beings tend to be routine-oriented. For instance, lack of regularity in daily mobility is linked to high levels of stress [31,32]. This change-averse behaviour leads people to favouring well-defined routines, which, in combination with stationarity [26], makes mobility trajectories quite regular [33]. Several factors such as work schedules and physiological processes, influence mobility-related decisions; for instance, daily necessities such as sleeping and eating influence activity schedules [34–39]. Conversely, disruptions to such routines can completely alter the mobility trajectories (as in the recent COVID-19 lockdown measures [40]), which can significantly alter mobility trajectories and therefore the associated levels of predictability [41].

Missing from extant measures of predictability are spatio-temporal constraints and the social embedding behind mobility regularities [42]—guessing that a person will be at home on Tuesday at 4.00 will most likely be a correct prediction. However, it may be much harder to know a person’s whereabouts during times in which they might not be bound by typical daily rhythms, on weekends, for example [43]. Additionally, predictability is computed from an asymptotic approximation of entropy based on a non-parametric estimator [44,45] that does not account for the periodic and rhythmic nature of travelling behaviours [46,47]. Finally, the metric does not provide insight into the generative mechanisms that underpin the observed regularities in mobility [19].

To overcome these limitations, we conduct studies on three location-based social networks (LBSNs) that contain location trajectories for all the users that participate in such networks. By location trajectories, we mean visitations made by individuals throughout the history of the data, represented as a vector whose elements correspond to a temporal sequence of alphabets with each alphabet corresponding to a unique labelling for the given location. Our work proposes a new perspective to mobility predictability that accounts for these missing factors, particularly connecting the observed regularities in mobility patterns to human circadian/semi-circadian routines and the types of locations they visit. Furthermore, we study the temporal variations of predictability, unveiling structural patterns in their frequency and time components, which we refer to as *predictability states*. Our results suggest that in addition to the daily routines, regularities in mobility predictability are also marked by periods of approximately 12 and 6 h, which could correspond to the second and fourth harmonics of internal circadian rhythm. These findings could suggest that factors governing mobility-related decisions are also influenced by internal biological cycles beyond sleeping and feeding needs. This is corroborated by the predominance of 12 h periods over other cycles governed by sleep/work/study routines, such as the 8 and 4 h cycles. Additionally, we show the role of a location-based context, demonstrating heterogeneities in mobility profiles based on the types of locations that people visit. The importance of this is demonstrated through agreement between the true distribution—calculated from the full set of visitation trajectories—and a simple linear model that takes only the frequency of visiting a location type as input. Taken together our results indicate that uncertainty and predictability in mobility patterns should be considered as a transient *state* of the individuals that is heavily influenced by their temporal and activity-location contexts.

## 2. Results

### 2.1. Datasets

We use data from three different LBSN services. Brightkite and Gowalla were two popular social networking sites that existed from 2007 until 2012. Weeplaces was a website where users could upload their check-in activities from other social network services (e.g. Facebook Places, Foursquare). These datasets contain users’ check-in activity including user identification, location coordinates (i.e. latitude and longitude) and the time-stamp of the logged activity. Additionally, the Weeplaces dataset contains a description (i.e. the category) of the locations (e.g. nightlife, outdoors). The details of the data are listed in table 1. For each individual in the dataset, we convert their check-in activity to trajectories described as a time series of the form

**Table 1.** Data from location-based social networking sites.

dataset	users	records	period
Brightkite [48]	58228	4491143	April 2008–October 2010
Gowalla [49]	107002	6405492	February 2009–October 2010
Weeplaces [50]	15799	7658368	November 2003–June 2011


X={x(1),x(2),…,x(T)},

where x(t)∈V is a location and V is the set of all visited locations by that individual. For more details on the data, refer to electronic supplementary materials, §S1, table S1 and figures S1– S6.

To compute the time-dependent predictability, we segment each user’s trajectory into non-overlapping windows of a specified size (1 h, for instance). The window size can be adjusted based on the research question. For each window (e.g. Monday at 1.00–2.00), we calculate the predictability based on the user’s location sequence within that window. This process is repeated for all windows across all users. Finally, we average the predictability scores across users for each specific time window (e.g. all users on Monday at 1.00) to obtain the population-level predictability for that time frame.

To analyse the effect of temporal window of observations, we segment each user’s entire trajectory for a defined period (e.g. a week with 168 h) into non-overlapping windows of the chosen size. This creates multiple ‘bins’ per user—168 bins for a 1 h window, 84 bins for 2 h windows and so on. We calculate a predictability score for each individual bin within a user’s trajectory. Averaging the individual bin scores for each user, results in a single predictability score that reflects their overall predictability across the chosen window size. For more details of the procedure, cf. electronic supplementary materials, §S1.3 and figure S7.

### 2.2. Time-independent uncertainty and predictability

**Figure 1.**
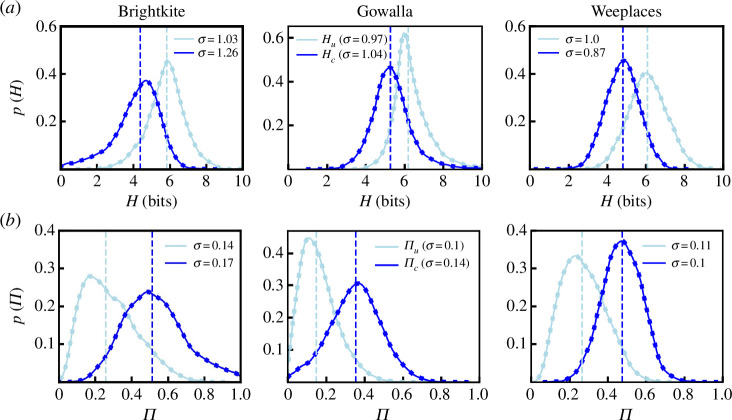
Time-independent entropy and predictability. (*a*) The entropy accounting for only visitation frequencies $H_{u}$ and incorporating the temporal sequence of visitations $H_{c}$. (*b*) The corresponding predictability values were calculated through equation (2.3). In all cases, accounting for the sequence reduces uncertainty from 6 to 5 bits and increases predictability from 20% to 40%. The vertical dashed lines represent the medians for each distribution while the values for the standard deviation σ are shown in the legends.

We begin our analysis by examining the information contained in the location trajectories of all individuals in each of the datasets. When accounting for only the frequency of location visitations, the degree of uncertainty in capturing the future locations of a trajectory given past observations is encoded in the Shannon entropy (measured in bits)


(2.1) $$H_{u}=-\sum_{i\in V}p(x)\log_{2}p(x),$$

where p(x) is the probability to visit the location x. The subscript refers to the fact that this is the uncorrelated entropy, given that no information on the sequence of location visits is considered. Accounting for both the visitation frequency and the temporal sequence of location visits, we use a non-parametric estimator [44,45] termed the entropy rate (Lempel–Ziv algorithm), given by the expression


(2.2) $$H_{c}=\frac{N\log_{2}N}{\sum_{i=1}^{N}\Lambda_{i}},$$

where N is the number of moves made by an individual (not necessarily to distinct locations), $\Lambda_{i}$ is the length of the shortest trajectory sub-sequence beginning at position i not seen previously. In the absence of any structure in the sequence, equation (2.2) reduces to equation (2.1). In figure 1*a*, we plot the results for the datasets finding that in all cases $H_{u}\approx 6$ bits and $H_{c}\approx 5$ bits. This indicates that accounting for the temporal sequence reduces the possible space of future location visits from $2^{6}$ to $2^{5}$ possible locations indicating the presence of temporal correlations in visits between locations.

The entropy rate can be converted to a measure of predictability Π using Fano’s inequality [51] to define the upper bound of how often an ideal predictive algorithm can correctly guess the next location visit, given prior history. This is calculated by inverting


(2.3) $$H_{u,c}\leq B(\Pi_{u,c})+(1-\Pi_{u,c})\log_{2}(S-1),$$

where S is the number of *distinct locations visited* and B(x) is the binary entropy function capturing the entropy of a simple Bernoulli trial (in this case achieving maximal predictability or not). The metric mathematically bounds the performance of all real predictive methods given an information sources inferred uncertainty. The corresponding results are shown in figure 1*b* and mirror the trends seen for the entropy; including information about the sequence of location visits, over and above the frequency of visiting those locations, increases the predictability from approximately 20% to 40%. In what is to follow we will interpolate between $\Pi_{u}$ and $\Pi_{c}$ based on the nature of the analysis.

### 2.3. Time-dependent predictability

Human activity routines are characterized by temporal regularities with time and frequency components. For instance, usual working hours tend to recur every 24 h (i.e. frequency) with changes during the weekends (i.e. time). This temporal regularity is reflected in the user check-in activity patterns observed in electronic supplementary material, figure S4. Thus, it is reasonable to believe that mobility regularities in both time and frequency domains should manifest themselves in the predictability profile of an individual. Thus, in order to extract the temporal variation of the predictability, instead of considering the complete visitation sequence of individuals, we analyse individuals at different moments of the week. Specifically, we split the trajectory of each individual into time slots representing the time of the week. We create 168 slots (i.e. 24 h × 7 days of the week) and define $X_{t=t_{0}}$ as a random variable representing the places that an individual visits at the time slot $t=t_{0}\in [1,\ldots ,168]$.

**Figure 2.**
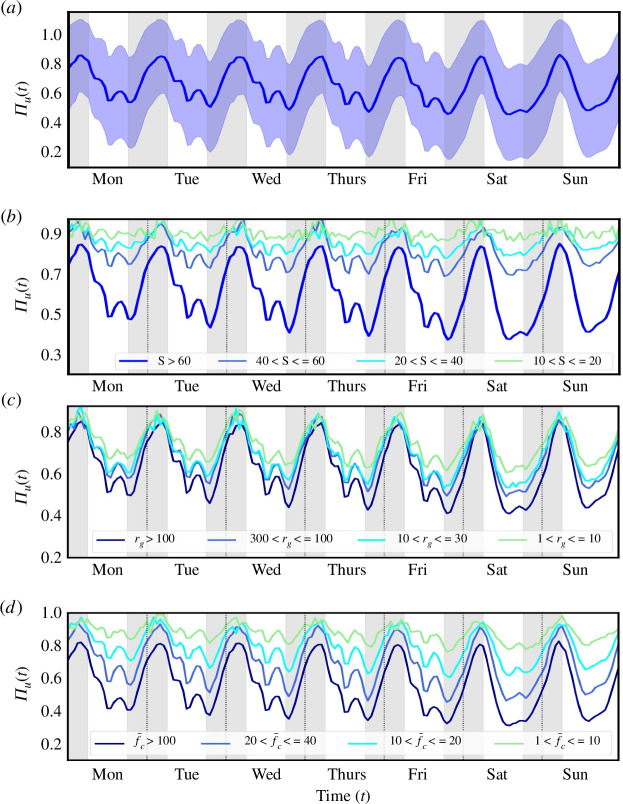
Time-dependent predictabilities. (*a*) The uncorrelated predictability $\Pi_{u}$, disaggregated with respect to time for Weeplaces. The trajectory of each individual is split into time slots representing the time of the week. We create 168 slots (i.e. 24h×7 days of the week) and define $X_{t=t_{0}}$ as a random variable representing the places that an individual visits at the time slot $t=t_{0}\in [1,\ldots ,168]$. The shaded area depicts the standard deviation (±σ) around the mean value, which is calculated for each hour. (*b*) The temporal predictability disaggregated with respect to the number of unique locations visited S. (*c*) Now disaggregated with respect to geographical coverage as measured by the radius of gyration $r_{g}$ in units of kilometres. (*d*) Finally, disaggregated with respect to the average frequency of monthly check-ins ${\bar{f}}_{c}$. Across all users, we see daily peaks (4.00 to 5.00) and secondary peaks (12.00 to 17.00) of predictability throughout the time series.

Given the shorter trajectories in a single-hour window, the temporal correlations are less important, and, consequently, we plot the uncorrelated predictability $\Pi_{u}$. The results are shown in figure 2*a*, where we plot the time-series of $\Pi_{u}(t)$ for Weeplaces—the corresponding plots for Brightkite and Gowalla are shown in electronic supplementary materials, figures S8 and S9. As the figure indicates, when disaggregating with respect to time, the predictability exhibits considerable variation both from day to day as well as within a given day. For instance, we see a progressive decrease in predictability from Monday to Friday, which then picks up again and peaks on the weekend. Within a day there is considerable variation, with the predictability of an individual fluctuating between 50% and 85%, with individuals being more predictable at night (the standard deviation is shown as the shaded region in figure 2*a* and is consistent with the range shown in figure 1*b*). Finally, we see a clear 24 h periodicity in the predictability profile with high predictability periods during evenings and at night (peaking around 4.00 to 5.00) and troughs at midday. This feature is seen across all three datasets.

We note that as in other human facets, human mobility and social interactions tend to exhibit a high degree of heterogeneity. If on one hand, most people use their mobile phones a few times a day, some users, on the other hand, make hundreds of calls a day. Some individuals are more active in the sense of travelling more frequently, to a diversity of locations, as well as travelling longer distances. One would expect this activity context to influence the predictability profiles in different ways. To test for this effect we disaggregate the data in terms of three different metrics of activity: number of unique locations visited S; geographical coverage measured by the *radius of gyration* $r_{g}$ [20]; and monthly recurrent frequency by averaging the number of check-ins per month ${\bar{f}}_{c}$. The electronic supplementary material, figure S1 shows the distribution of these quantities for all the datasets, indicating a right-skewed heavy-tailed distribution for all three measures in line with previous observations [20,25].

The influence of the observed heterogeneity in the activity metrics on the predictability is plotted in figure 2*b–d*. First, we note that the temporal trends are maintained even while disaggregating with respect to the activity metrics. However, the range in predictability varies with respect to the levels of activity. For instance, those who visit between 10 and 20 unique locations have an effectively flat temporal profile across the days of the week, as well as within a day ($\Pi_{u}\approx 90\%$). In contrast, those who visit more than 60 unique locations show much more temporal variability with lower levels of predictability ($50\%\leq \Pi_{u}\leq 80\%$). Additionally one sees a sharp drop in predictability when comparing populations visiting less than 60 locations with those visiting more than this number. A similar trend is seen for geographical coverage, where once again a flat trend is exhibited by populations venturing not more than 10 km from their residence, whereas those travelling more than 100 km, experience the same variability as those visiting more than 60 unique locations. Now one sees a sharp drop in $\Pi_{u}$ when comparing populations travelling more than 100 km with those that travel less. Finally, the trend is also mirrored when measuring the frequency of check-ins ${\bar{f}}_{c}$, the difference being the absence of any sharp drops in $\Pi_{u}$ with a more smooth decrease between the segmented populations. Electronic supplementary material, figures S8 and S9 indicate the same trends for Brightkite and Gowalla. Across all datasets, the predictability decreases with more diversity in activity (as measured by S, $r_{g}$ and ${\bar{f}}_{c}$). That is, it is harder to predict the mobility of users with more *diverse* routes irrespective of whether the diversity is measured in terms of the number of unique locations visited, the geographical area explored, or the amount of monthly data (traces). Taken together the results indicate an unexplored facet of uncertainty and predictability; stating that ‘an individual is 80% predictable’ must be interpreted in an *averaged* sense. Missing from this is the instantaneous changes in a person’s *predictability state* over time.

**Figure 3.**
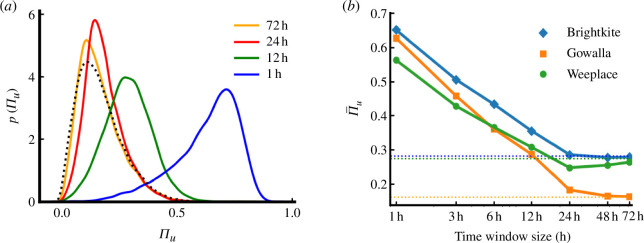
The effect of temporal windows of observation. (*a*) The distribution of $\Pi_{u}$ as a function of window size for Gowalla. The dashed curve represents the baseline distribution of $\Pi_{u}$ when taking into account the full trajectory of individuals. (*b*) The modes of the distributions are plotted as a function of window size for all three datasets. Horizontal dashed lines indicate the saturated value of $\Pi_{u}$.

Indeed, one would also expect this to vary with respect to the observation window of an individual’s trajectory. For instance, if we observe a user only for an hour, they are likely to visit only a few locations. Given the lack of diversity in routes, the predictability should be high (as indicated by figure 2). Conversely, the longer the observation window, the more the number of locations visited, and therefore the predictability necessarily should decrease. It is interesting to consider whether there is a saturation in this decrease in $\Pi_{u}$. In figure 3*a*, we plot the distribution of $\Pi_{u}$ as a function of the temporal window of observations for Gowalla, ranging from 1 to 72 h windows. As a baseline, the curve for the predictability considering the full set of trajectories (absent any window) is shown as a dashed line. As the time window gets smaller, the average predictability increases as expected. This is because we move from daily bins (each bin representing a full day of 24 h) to hourly bins (each bin representing a single hour within a day). In figure 3*b*, we plot the mode of the distributions as a function of the window size finding a saturation in the curve at 24 h for all three datasets.

### 2.4. Temporal and frequency modes in predictability

**Figure 4.**
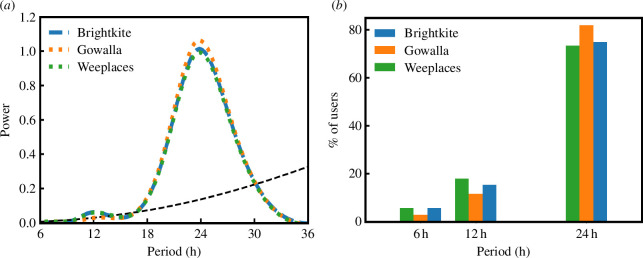
Temporal modes of predictability. (*a*) Estimated global wavelet power spectrum showing peaks at 24 hours (circadian) and 12 h (circasemidian) as well as a non-significant peak at 6 h. The dashed black line represents the statistical significance of the mode as measured by electronic supplementary material, equation (S3). (*b*) Stacked bar chart for the most strong component period (24, 12 and 6 hours) of each individual.

The temporal variation of the predictability seen in figure 2 suggests the possible presence of additional frequency modes in addition to the clear 24 h periodicity. To uncover this, one can use the continuous wavelet transform to describe the regularities in the time series of the individuals [52,53]. Wavelet analysis reveals the frequency components of signals just like the Fourier transform, but it also identifies where a certain frequency exists in the temporal or spatial domain (see electronic supplementary material, §S4 for details of the method). Figure 4*a* shows the wavelet power spectrum for each of the datasets (the dashed line indicates the statistical significance, electronic supplementary material, equation S3). Not surprisingly, the figure reveals the circadian period (approximately 24 h) as the most prominent component of the predictability regularity. Additionally, and somewhat surprisingly, the 12 h component is the second-strongest component (i.e. the circasemidian period). Given working schedules and sleeping cycles, one might have expected a mode around the 8 h period, however, the third-strongest component is centred approximately around the 6 h regime during the day, even though the signal is not statistically significant at the population level. We note that the power spectrum is essentially identical across all three datasets, indicating the robustness of these modes in determining the observed temporal variation in predictability. Conducting the wavelet analysis on individual-level data, one can represent the percentage of users in each dataset that have either the 24, 12 or 6 h mode as their strongest components. The results are shown in figure 4*b* indicating that across all datasets between 73% and 81% of users have the 24 h mode as their strongest component, 12–18% of users have instead the 12 h mode as their strongest component, whereas a negligible fraction have the 6 h mode.

### 2.5. Location context of predictability

Thus far we have investigated the temporal context of uncertainty in mobility behaviour. It stands to reason that there is also a location-based context that influences movement patterns. For example, it is plausible that people are more predictable about their workplace or residence, as compared with locations that represent leisure and entertainment activities such as visiting restaurants and museums. This is a function of those types of locations being less frequently visited as compared with those that are driven by the daily work schedules.

To investigate this, one can scan the sequence of trajectory visits and group each location into categories. Now that we are once again analysing the full sequence of trajectories, temporal correlations in the sequence come into play and we can do a comparative analysis of both the correlated and uncorrelated predictabilities. The Weeplaces dataset contains a standardized set of eight location tags: *Home/Work*, *Education*, *Entertainment*, *Food*, *Travel*, *Shops*, *Outdoors* and *Nightlife*. Restricting the analysis of trajectories to each location type, one can compare it with the baseline distributions for $\Pi_{u,c}$ when considering all types of locations. For instance, a context-dependent trajectory for food could be: *breakfast-place-A*, *lunch-place-B*, *coffee-place-C*, etc.; while a full baseline trajectory could be: *home-place-X*, *breakfast-place-A*, *work-place-Y*, *lunch-place-B*, *coffee-place-C*, *gym-place-Z*, etc.

**Figure 5.**
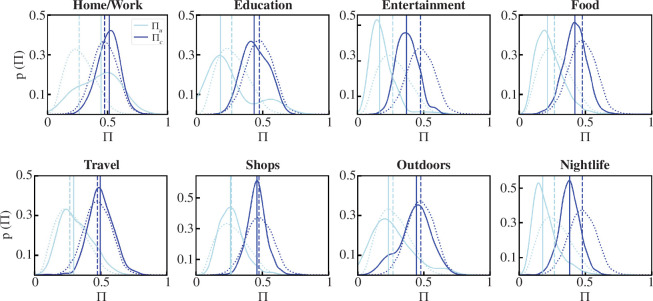
Location context of predictability. In all subplots, the dotted lines represent the distribution of $\Pi_{u,c}$ given their full trajectories for Weeplaces. Solid lines represent distributions for the same individuals but when limiting their trajectories to locations of distinct types. The vertical dashed lines correspond to the median values.

The results are shown in figure 5 with the baseline distribution shown as a dashed line. The figure clearly indicates the role of a spatial context in the distributions for both $\Pi_{c}$ and $\Pi_{u}$. For instance, as expected, activities related to *Home/Work, Travel, Shops* are more predictable than the baseline; *Education*, *Outdoors* essentially mirror the baseline, whereas activities corresponding to *Food*, *Entertainment*, *Nightlife* have peaks markedly below the baseline.

The observed difference in predictability as a function of location-context behoves us to investigate the extent to which the frequency of which particular location types are visited has a bearing on the overall distribution of $\Pi_{c}$. Note this is different from merely considering the frequency distribution of visiting a *particular location* which is the primary input to equation (2.1). Instead, here we coarse-grain these locations into categories and investigate whether this feature can be a good estimator for $P(\Pi_{c})$. To that effect, we consider a linear regression model by using the relative frequency that an individual stayed at places from each category as an input. For instance, we investigate whether knowing that a person goes shopping often, informs us about this person’s overall predictability. We train the model with the calculated predictability as an independent variable and the relative frequencies of the categories as the dependent variable.

**Figure 6.**
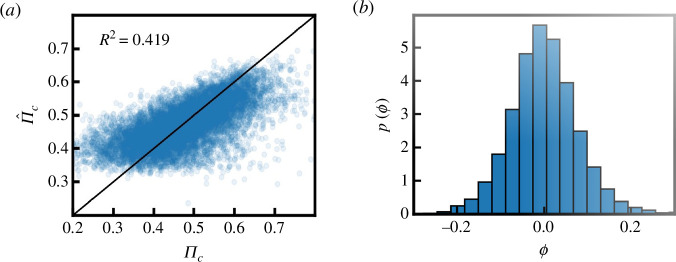
Estimating predictability from location context. (*a*) The estimated predictability ${\hat{\Pi }}_{c}$ using a linear model with frequency of visiting location types as input, plotted against the true distribution of $\Pi_{c}$ in the Weeplaces dataset ($R^{2}=0.419$). (*b*) The residuals are normally distributed and centred around zero.

Using this linear model, we estimate the predictability denoted by $P({\hat{\Pi }}_{c})$ (calculated from coarse-grained trajectories, e.g. 80% home/work, 18% food, 1% nightlife, etc.) in figure 6*a* and plot it against the true distribution $P(\Pi_{c})$ (calculated from the full trajectories over multiple days, e.g. home, restaurant, work, restaurant, etc.). As the figure indicates, this simple model does a reasonable job of estimating the true distribution ($R^{2}=0.419$) with the residuals being normally distributed and centred around zero (figure 6*b*). (The coefficients of the model are shown in electronic supplementary material, table S2). Our results highlight two key points. First, the linear model’s effectiveness as an approximation to the true distribution suggests the importance of location contexts. Second, this implies that capturing coarse-grained information about individuals, such as location types and visit frequencies, is sufficient to understand the general trends in mobility uncertainty, without resort to the full set of fine-grained mobility trajectories.

## 3. Discussion

Meeting a citizenry’s needs require governments, industries and other stakeholders to be able to plan for demands (e.g. hospital admissions, public transportation and store opening times). The predictability of human movement is at the heart of planning, hence more accurate modelling should inform better decision-making in terms of public policy. Extant research models uncertainty and predictability in mobility trajectories as aggregated value for individual considering their full set of mobility trajectories as an input. This neglects the spatio-temporal factors influencing mobility, such as the temporal variations for different times of the day and days of the week, the levels of activity as well as the types of locations visited. As our results indicate, the predictability is greatly influenced by the window of observation, the length of the observation, the diversity of activities undertaken by an individual, as well as the location of the individual. Consequently, a more accurate way of modelling predictability is to consider it as a transient state, while taking into account the activity context.

The role of location context has some practical considerations. As our findings demonstrate, using coarse-grained location types and measuring visit frequencies provides a reasonable proxy for the predictability extracted from mobility trajectories. While traditional methods require finely grained data with frequent sampling, this approach offers a simpler and potentially more efficient alternative. Indeed, typically high-resolution mobility data are hard to come by and are restricted to only a few regions in the world. Our results show that much can be learnt even with low-resolution information. On the other hand, there is increasing access to higher-resolution data, such as the ones being collected as part of ‘track-and-trace’ systems in certain countries. In such instances, our framework of combining mobility traces with activity context can lead to more accurate characterizations. Indeed, many governments and companies (as part of ‘data-for-good’ efforts) are starting to open their datasets to scientists, which will naturally lead to better urban analytics, including human dynamics modelling.

The main implication of this work is that planning of activities related to human mobility (e.g. city events, epidemic modelling and road maintenance) needs to consider time–space variations of individual activities. Furthermore, during periods of restrictions, such as the COVID-19 pandemic, the understanding and characterization of these time–spatial variations can aid governments to make the correct decisions. In 2020 and 2021, many governments imposed curfew/lockdown measures on citizens after certain hours (e.g. Spain, Colombia) in a ‘blanket’ way. Effective curfews depend on the time and the location, and the consideration of such variations can lead to a better approach where not all areas are treated equally. Using predictability as a state and using the states for planning could lead to more just/equitable outcomes.

### 3.1. Limitations

Our work of course has limitations; given the analysis was conducted on a particular type of dataset (LBSNs) that vary in their spatio-temporal resolution, geographical coverage and sampling rates. It will be instructive to conduct this analysis in different regions of the world and using other sources of mobility information such as censuses, GPS data and call data records. Nevertheless, given that we conducted the analysis on three different datasets, finding similar trends points towards the results holding up to scrutiny in different settings.

Our analysis relies on the assumption that check-in data comprehensively represents all user activities throughout the day or week. In reality, limitations in data collection methods and potential user participation bias may affect this assumption. Additionally, while our datasets capture spatial information (latitude and longitude), the data reflects app usage rather than detailed activity types. Check-ins may not distinguish between specific activities at a given location, such as a work meeting, lunch break, or shopping trip. These factors call for cautious interpretation of the results and highlight the need for further research that incorporates additional data sources or methods to capture a more nuanced picture of user activity.

## Data Availability

*Brightkite and Gowalla data*: These LBSN datasets are publicly available from the Stanford Network Analysis Project (SNAP) database. We accessed them using their respective accession codes: Brightkite [48] and Gowalla [49]. Both datasets are cited Brightkite and Gowalla within the paper. *Weeplaces data*: This LBSN dataset is owned by Yong Liu and can be downloaded from their website: [50]. We have cited this data source Weeplaces within the paper. Supplementary material is available online [54].
